# 2-(Naphthalen-1-yl)-1-phenyl-1*H*-benzimid­azole benzene hemisolvate

**DOI:** 10.1107/S160053681303331X

**Published:** 2013-12-14

**Authors:** N. Srinivasan, A. Thiruvalluvar, S. Rosepriya, S. M. Prakash, R. J. Butcher

**Affiliations:** a Research and Development Center, Bharathiar University, Coimbatore 641 046, Tamilnadu, India; b Department of Chemistry, S.K.P. Engineering College, Thiruvannamalai 606 611, Tamilnadu, India; c Postgraduate Research Department of Physics, Rajah Serfoji Government College (Autonomous), Thanjavur 613 005, Tamilnadu, India; d Department of Chemistry, Annamaliar College of Engineering, Mudaiyur 606 902, Tamilnadu, India; e Department of Chemistry, Howard University, 525 College Street NW, Washington, DC 20059, USA

## Abstract

In the title compound, C_23_H_16_N_2_·0.5C_6_H_6_, the benzimidazole unit [maximum deviation = 0.0258 (6) Å] and the naphthalene ring system [maximum deviation = 0.0254 (6) Å] are both essentially planar and make a dihedral angle of 61.955 (17)°. The imidazole ring makes dihedral angle of 61.73 (4)° with the phenyl ring. An intra­molecular C—H⋯N hydrogen bond generates an *S*(6) ring motif. In the crystal, seven weak C—H⋯π inter­actions involving the fused ring system, the benzene solvent mol­ecule, the imidazole phenyl rings are observed, leading to a three-dimensional architecture.

## Related literature   

For linear and non-linear optical properties and the thermal stability of benzimidazole-based chromophores, see: Cross *et al.* (1995 ▶). For imidazole as a component of vitamin B_12_, purine and caffeine, see: Brown (2005 ▶). For commercial and therapeutic applications of substituted benzimidazole derivatives, see: Spasov *et al.* (1999 ▶). For related crystal structures, see: Jayamoorthy *et al.* (2012 ▶, 2013 ▶); Rosepriya *et al.* (2011 ▶). For hydrogen-bond motifs, see: Bernstein *et al.* (1995 ▶).
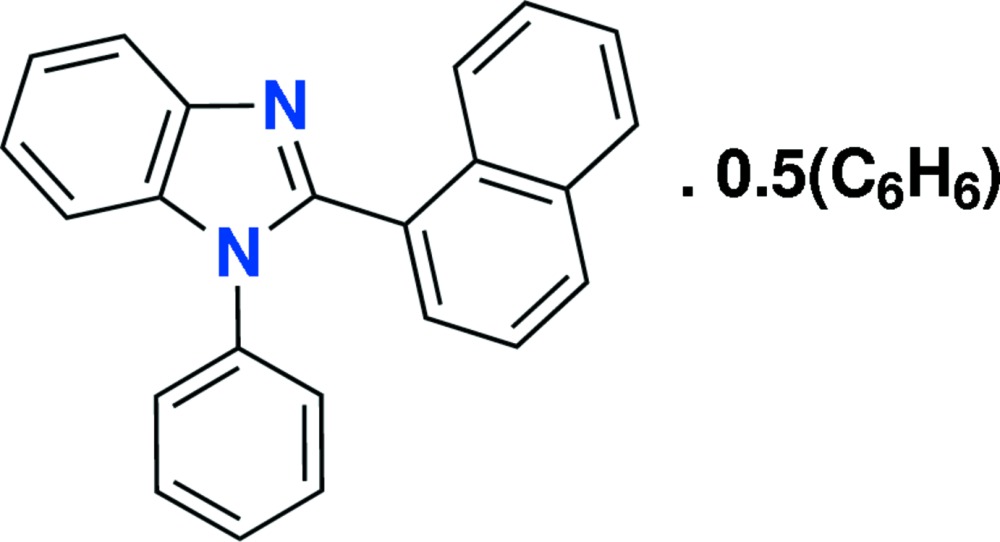


## Experimental   

### 

#### Crystal data  

C_23_H_16_N_2_·0.5C_6_H_6_
*M* *_r_* = 359.43
Triclinic, 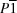

*a* = 8.5529 (3) Å
*b* = 9.4517 (3) Å
*c* = 11.8936 (3) Å
α = 86.334 (2)°
β = 89.838 (2)°
γ = 75.051 (3)°
*V* = 926.94 (5) Å^3^
*Z* = 2
Mo *K*α radiation
μ = 0.08 mm^−1^
*T* = 123 K
0.72 × 0.59 × 0.42 mm

#### Data collection  

Agilent Xcalibur Ruby Gemini diffractometer
Absorption correction: analytical [*CrysAlis PRO* (Agilent, 2012 ▶), using a multifaceted crystal model (Clark & Reid, 1995 ▶)] *T*_min_ = 0.963, *T*_max_ = 0.977
57784 measured reflections
12045 independent reflections
9086 reflections with *I* > 2σ(*I*)
*R*_int_ = 0.063

#### Refinement  

*R*[*F* ^2^ > 2σ(*F* ^2^)] = 0.057
*wR*(*F* ^2^) = 0.160
*S* = 1.05
12045 reflections
253 parameters
H-atom parameters constrained
Δρ_max_ = 0.48 e Å^−3^
Δρ_min_ = −0.42 e Å^−3^

### 

Data collection: *CrysAlis PRO* (Agilent, 2012 ▶); cell refinement: *CrysAlis PRO*; data reduction: *CrysAlis PRO*; program(s) used to solve structure: *SHELXS2013* (Sheldrick, 2008 ▶); program(s) used to refine structure: *SHELXL2013* (Sheldrick, 2008 ▶); molecular graphics: *ORTEP-3 for Windows* (Farrugia, 2012 ▶) and *PLATON* (Spek, 2009 ▶); software used to prepare material for publication: *SHELXL2013* and *PLATON* (Spek, 2009 ▶).

## Supplementary Material

Crystal structure: contains datablock(s) global, I. DOI: 10.1107/S160053681303331X/jj2179sup1.cif

Structure factors: contains datablock(s) I. DOI: 10.1107/S160053681303331X/jj2179Isup2.hkl

Supporting information file. DOI: 10.1107/S160053681303331X/jj2179Isup3.cdx

Supporting information file. DOI: 10.1107/S160053681303331X/jj2179Isup4.cml

Additional supporting information:  crystallographic information; 3D view; checkCIF report

## Figures and Tables

**Table 1:** Hydrogen-bond geometry (Å, °) *Cg*1, *Cg*2, *Cg*3, *Cg*4 and *Cg*8 are the centroids of the N1/C2/N3/C9/C8 imidazole ring, the C4–C9 fused benzene ring, the C11–C16 phenyl ring, the C21–C24,C30/C29 fused benzene ring and the C1*A*,C2*A*,C3*A*′,C1*A*′,C2*A*′,C3*A* benzene ring, respectively.

*D*—H⋯*A*	*D*—H	H⋯*A*	*D*⋯*A*	*D*—H⋯*A*
C28—H28⋯N3	0.95	2.61	3.2113 (10)	121
C7—H7⋯*Cg*4^i^	0.95	2.75	3.6019 (8)	150
C15—H15⋯*Cg*8^ii^	0.95	2.99	3.6981 (9)	132
C15—H15⋯*Cg*8^iii^	0.95	2.99	3.6981 (9)	132
C22—H22⋯*Cg*1^iv^	0.95	2.91	3.6478 (8)	136
C24—H24⋯*Cg*3^v^	0.95	2.76	3.4888 (9)	134
C26—H26⋯*Cg*2^iii^	0.95	2.87	3.5801 (9)	133
C27—H27⋯*Cg*1^iii^	0.95	2.97	3.7258 (8)	137

Symmetry codes: (i) 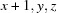; (ii) 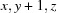; (iii) 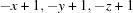; (iv) 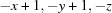; (v) 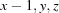.

**Fig. 1.**
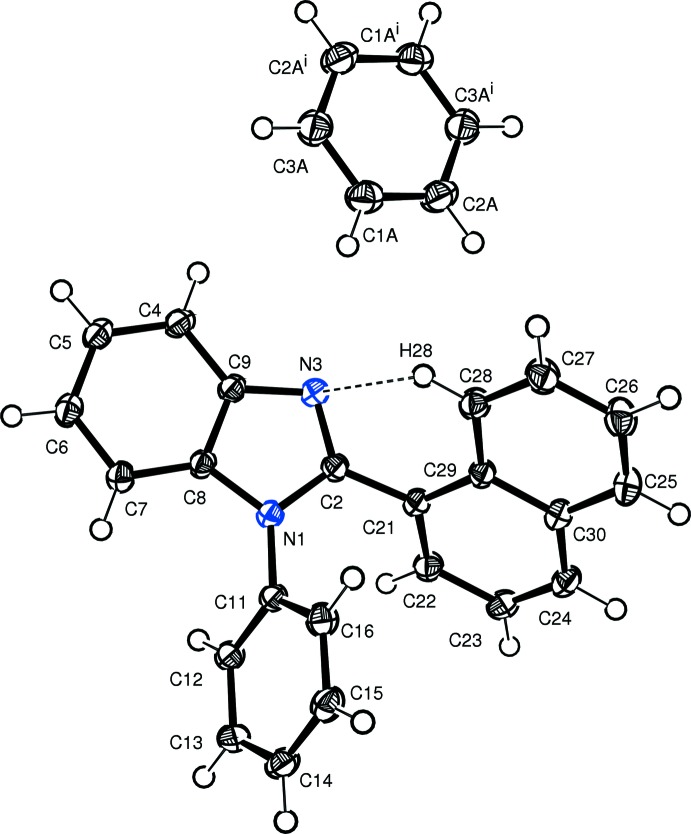
The molecular structure of the title compound, with displacement ellipsoids drawn at the 50% probability level. H atoms are shown as small spheres of arbitrary radius. The dashed line indicates the intramolecular C—H···N hydrogen bond. Symmetry code: (i) -x + 1, -y, -z + 1.

**Fig. 2.**
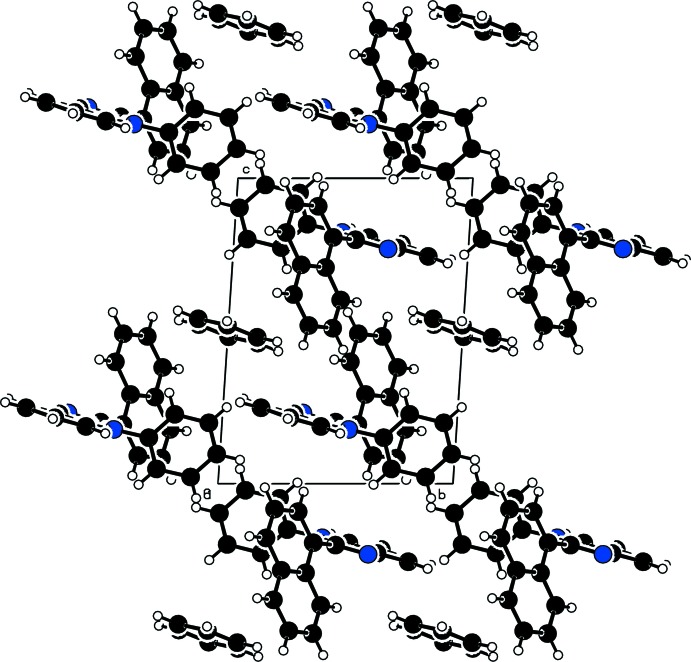
The packing of the title compound, viewed along the a axis.

**Fig. 3.**
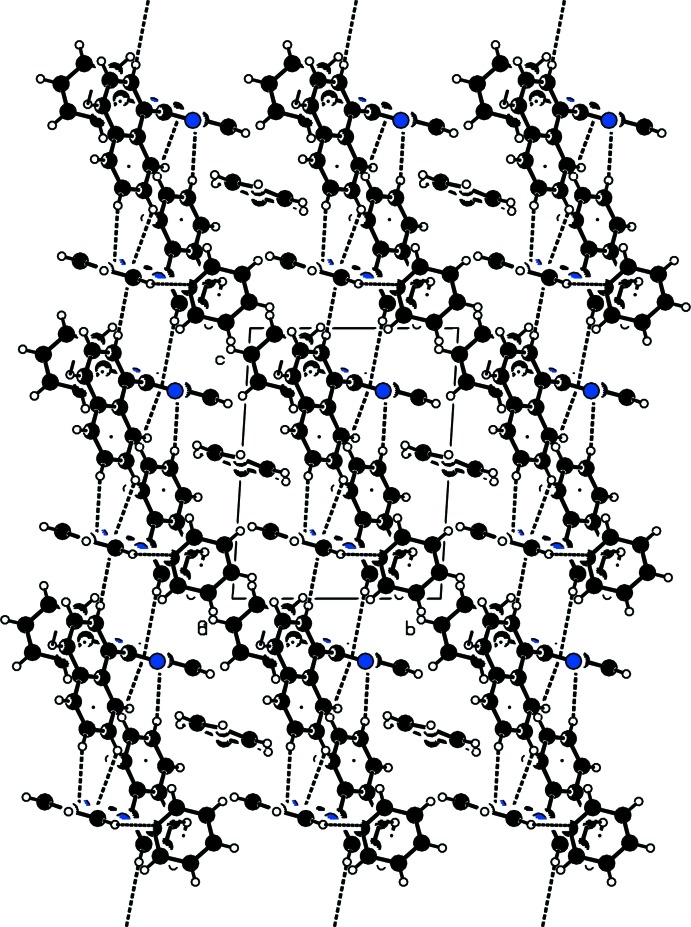
Part of the crystal structure of compound, showing the formation of C—H···π interactions. Interactions involving benzene solvent Cg8 are not shown.

## supplementary crystallographic information

### 1. Comment

Benzimidazole based chromophores have received increasing attention due to their distinctive linear, non-linear optical properties and also due to their excellent thermal stability in guest-host systems (Cross *et al.*, 1995). They are a component of vitamin B_12_ (Brown, 2005) and are related to the DNA base purine and the stimulant caffeine. Substituted benzimidazole derivatives have found commercial applications in veterinarian medicine as anthelmintic agents and in diverse human therapeutic areas as an antiulcer and antihistaminic (Spasov *et al.*, 1999). Therefore, the preparation of benzimidazoles has gained considerable attention in recent years. Jayamoorthy *et al.*, (2012, 2013) and Rosepriya *et al.*, (2011) have reported closely related structures of benzimidazole derivatives. We are interested to use 2-(naphthalen-1-yl)-1-phenyl-1*H*-benzimidazole as ligand to study its photophysical properties.

The assymmetric unit of the title compound, C_23_H_16_N_2_·0.5C_6_H_6_, (Fig. 1), contains a 2-(naphthalen-1-yl)-1-phenyl-1*H*-benzimidazole molecule and a hemibenzene solvent molecule. The benzimidazole unit is essentially planar [maximum deviation of -0.0258 (6) Å for C8]. The naphthalene unit is also essentially planar [maximum deviation of -0.0254 (6) Å for C23]. The benzimidazole unit makes dihedral angle of 61.955 (17)° with the naphthalene unit. The imidazole ring makes dihedral angle of 61.73 (4)° with the phenyl group attached to N1.

An intramolecular C28—H28···N3 hydrogen bond (Fig. 1) generates an S(6) ring motif (Bernstein *et al.*, 1995). The packing of the title compound, viewed along the *a* axis is shown in Fig. 2. In the crystal, seven weak C7—H7···π interactions involving the fused benzene ring, C15—H15···π interactions involving the solvent benzene ring, C22—H22···π interaction involving the imidazole ring, C24—H24···π interaction involving the phenyl ring, C26—H26···π interaction involving the fused benzene ring, C27—H27···π interaction involving the imidazole ring, are observed, leading to a three dimensional architecture (Fig. 3, Table 1).

### 2. Experimental

To the pure *N*-phenyl-*o*-phenylenediamine (17 mmol, 3.128 g) in ethanol (10 ml), napthaldehyde (17 mmol, 1.9 ml) and ammonium acetate (3 g) was added about 1 h by maintaining the temperature at 353 K. The reaction mixture was refluxed for 48 hrs and the completion of reaction was monitored by TLC, finally the reaction extracted by dichloromethane. The solid separated was purified by column chromatography using benzene as the eluent. Yield: 2.65 g (50%). The compound was dissolved in benzene and ethyl acetate (9:1) mixture and allowed to slow evaporation for two days, to obtain crystals suitable for X-ray diffraction studies.

### 3. Refinement

The H atoms were positioned geometrically and allowed to ride on their parent atoms, with C—H = 0.95 Å. *U*_iso_(H) = *1.2U*_eq_(C).

### Crystal data

**Table:**

C_23_H_16_N_2_·0.5C_6_H_6_	*Z* = 2
*M**_r_* = 359.43	*F*(000) = 378
Triclinic, *P*1	*D*_x_ = 1.288 Mg m^−^^3^
Hall symbol: -P 1	Melting point: 392 K
*a* = 8.5529 (3) Å	Mo *K*α radiation, λ = 0.71069 Å
*b* = 9.4517 (3) Å	Cell parameters from 15125 reflections
*c* = 11.8936 (3) Å	θ = 3.0–41.0°
α = 86.334 (2)°	µ = 0.08 mm^−^^1^
β = 89.838 (2)°	*T* = 123 K
γ = 75.051 (3)°	Prism, colourless
*V* = 926.94 (5) Å^3^	0.72 × 0.59 × 0.42 mm

### Data collection

**Table:**

Agilent Xcalibur Ruby Gemini diffractometer	12045 independent reflections
Radiation source: Enhance (Mo) X-ray Source	9086 reflections with *I* > 2σ(*I*)
Graphite monochromator	*R*_int_ = 0.063
Detector resolution: 10.5081 pixels mm^-1^	θ_max_ = 41.1°, θ_min_ = 3.0°
ω scans	*h* = −15→15
Absorption correction: analytical [*CrysAlis PRO* (Agilent, 2012), using a multifaceted crystal model (Clark & Reid, 1995)]	*k* = −17→17
*T*_min_ = 0.963, *T*_max_ = 0.977	*l* = −21→21
57784 measured reflections	

### Refinement

**Table:**

Refinement on *F*^2^	Primary atom site location: structure-invariant direct methods
Least-squares matrix: full	Secondary atom site location: difference Fourier map
*R*[*F*^2^ > 2σ(*F*^2^)] = 0.057	Hydrogen site location: inferred from neighbouring sites
*wR*(*F*^2^) = 0.160	H-atom parameters constrained
*S* = 1.05	*w* = 1/[σ^2^(*F*_o_^2^) + (0.076*P*)^2^ + 0.0921*P*] where *P* = (*F*_o_^2^ + 2*F*_c_^2^)/3
12045 reflections	(Δ/σ)_max_ < 0.001
253 parameters	Δρ_max_ = 0.48 e Å^−^^3^
0 restraints	Δρ_min_ = −0.42 e Å^−^^3^

### Special details

**Table:**

Geometry. Bond distances, angles *etc*. have been calculated using the rounded fractional coordinates. All su's are estimated from the variances of the (full) variance-covariance matrix. The cell e.s.d.'s are taken into account in the estimation of distances, angles and torsion angles

### Fractional atomic coordinates and isotropic or equivalent isotropic displacement parameters (Å^2^)

**Table:**

	*x*	*y*	*z*	*U*_iso_*/*U*_eq_	
N1	0.67234 (7)	0.54147 (6)	0.17737 (5)	0.0196 (1)	
N3	0.58580 (7)	0.34162 (7)	0.23145 (5)	0.0218 (1)	
C2	0.54214 (8)	0.48307 (7)	0.20135 (6)	0.0197 (1)	
C4	0.86569 (9)	0.16857 (8)	0.25577 (6)	0.0230 (2)	
C5	1.02936 (9)	0.16196 (8)	0.24850 (6)	0.0241 (2)	
C6	1.08319 (9)	0.28681 (9)	0.21552 (6)	0.0237 (2)	
C7	0.97488 (9)	0.42165 (8)	0.18733 (6)	0.0222 (2)	
C8	0.81081 (8)	0.42675 (7)	0.19437 (6)	0.0194 (1)	
C9	0.75441 (8)	0.30379 (7)	0.22843 (6)	0.0200 (2)	
C11	0.66581 (8)	0.69261 (7)	0.15057 (5)	0.0190 (1)	
C12	0.72504 (9)	0.73392 (8)	0.04813 (6)	0.0217 (2)	
C13	0.71614 (9)	0.88153 (8)	0.02178 (6)	0.0247 (2)	
C14	0.64697 (9)	0.98608 (8)	0.09676 (7)	0.0253 (2)	
C15	0.58828 (9)	0.94403 (8)	0.19913 (7)	0.0259 (2)	
C16	0.59863 (9)	0.79665 (8)	0.22698 (6)	0.0233 (2)	
C21	0.37491 (8)	0.57825 (7)	0.19356 (6)	0.0194 (2)	
C22	0.31678 (9)	0.65122 (8)	0.09166 (6)	0.0230 (2)	
C23	0.16256 (9)	0.75196 (9)	0.08347 (6)	0.0252 (2)	
C24	0.07012 (9)	0.78075 (8)	0.17773 (7)	0.0243 (2)	
C25	0.02654 (9)	0.73198 (9)	0.38062 (7)	0.0270 (2)	
C26	0.07884 (10)	0.65771 (10)	0.48214 (7)	0.0291 (2)	
C27	0.23194 (10)	0.55553 (10)	0.49096 (6)	0.0271 (2)	
C28	0.32981 (9)	0.52890 (8)	0.39875 (6)	0.0225 (2)	
C29	0.27828 (8)	0.60281 (7)	0.29210 (5)	0.0187 (2)	
C30	0.12426 (8)	0.70669 (8)	0.28362 (6)	0.0210 (2)	
C1A	0.55334 (12)	0.12597 (10)	0.47928 (7)	0.0319 (2)	
C2A	0.39180 (12)	0.13022 (10)	0.46027 (8)	0.0329 (2)	
C3A	0.66115 (11)	−0.00416 (11)	0.51904 (7)	0.0315 (2)	
H4	0.83010	0.08400	0.27860	0.0276*	
H5	1.10668	0.07119	0.26616	0.0289*	
H6	1.19612	0.27882	0.21245	0.0285*	
H7	1.01084	0.50599	0.16443	0.0266*	
H12	0.77109	0.66228	−0.00334	0.0261*	
H13	0.75746	0.91078	−0.04763	0.0297*	
H14	0.63978	1.08678	0.07795	0.0303*	
H15	0.54107	1.01593	0.25013	0.0311*	
H16	0.56028	0.76719	0.29741	0.0279*	
H22	0.38129	0.63341	0.02635	0.0276*	
H23	0.12295	0.79973	0.01263	0.0302*	
H24	−0.03168	0.85133	0.17208	0.0292*	
H25	−0.07625	0.80110	0.37523	0.0323*	
H26	0.01210	0.67503	0.54642	0.0350*	
H27	0.26770	0.50460	0.56147	0.0325*	
H28	0.43288	0.46046	0.40632	0.0270*	
H1A	0.58997	0.21206	0.46507	0.0383*	
H2A	0.31790	0.21918	0.43320	0.0394*	
H3A	0.77144	−0.00685	0.53211	0.0378*	

### Atomic displacement parameters (Å^2^)

**Table:**

	*U*^11^	*U*^22^	*U*^33^	*U*^12^	*U*^13^	*U*^23^
N1	0.0184 (2)	0.0182 (2)	0.0204 (2)	−0.0019 (2)	0.0024 (2)	0.0005 (2)
N3	0.0201 (2)	0.0205 (2)	0.0231 (3)	−0.0031 (2)	0.0015 (2)	0.0016 (2)
C2	0.0188 (2)	0.0205 (3)	0.0184 (2)	−0.0031 (2)	0.0016 (2)	0.0005 (2)
C4	0.0238 (3)	0.0194 (3)	0.0233 (3)	−0.0013 (2)	0.0001 (2)	0.0004 (2)
C5	0.0226 (3)	0.0222 (3)	0.0236 (3)	0.0011 (2)	−0.0003 (2)	−0.0009 (2)
C6	0.0192 (3)	0.0260 (3)	0.0234 (3)	−0.0009 (2)	0.0011 (2)	−0.0025 (2)
C7	0.0197 (3)	0.0226 (3)	0.0230 (3)	−0.0034 (2)	0.0023 (2)	−0.0016 (2)
C8	0.0183 (2)	0.0191 (3)	0.0186 (2)	−0.0013 (2)	0.0016 (2)	−0.0009 (2)
C9	0.0199 (3)	0.0192 (3)	0.0190 (3)	−0.0021 (2)	0.0011 (2)	−0.0003 (2)
C11	0.0184 (2)	0.0184 (2)	0.0186 (2)	−0.0021 (2)	0.0010 (2)	0.0001 (2)
C12	0.0222 (3)	0.0213 (3)	0.0198 (3)	−0.0026 (2)	0.0024 (2)	0.0003 (2)
C13	0.0248 (3)	0.0240 (3)	0.0238 (3)	−0.0050 (2)	−0.0014 (2)	0.0050 (2)
C14	0.0230 (3)	0.0195 (3)	0.0315 (3)	−0.0032 (2)	−0.0053 (3)	0.0023 (2)
C15	0.0245 (3)	0.0214 (3)	0.0303 (3)	−0.0021 (2)	0.0003 (3)	−0.0062 (2)
C16	0.0242 (3)	0.0235 (3)	0.0215 (3)	−0.0045 (2)	0.0034 (2)	−0.0040 (2)
C21	0.0175 (2)	0.0196 (3)	0.0198 (3)	−0.0028 (2)	0.0003 (2)	0.0004 (2)
C22	0.0221 (3)	0.0252 (3)	0.0199 (3)	−0.0036 (2)	−0.0003 (2)	0.0019 (2)
C23	0.0241 (3)	0.0251 (3)	0.0238 (3)	−0.0032 (2)	−0.0043 (2)	0.0041 (2)
C24	0.0201 (3)	0.0219 (3)	0.0282 (3)	−0.0008 (2)	−0.0039 (2)	0.0000 (2)
C25	0.0202 (3)	0.0296 (3)	0.0287 (3)	−0.0011 (3)	0.0032 (2)	−0.0065 (3)
C26	0.0264 (3)	0.0355 (4)	0.0246 (3)	−0.0055 (3)	0.0067 (3)	−0.0059 (3)
C27	0.0273 (3)	0.0326 (4)	0.0199 (3)	−0.0054 (3)	0.0027 (2)	0.0001 (3)
C28	0.0211 (3)	0.0245 (3)	0.0201 (3)	−0.0030 (2)	0.0004 (2)	0.0004 (2)
C29	0.0167 (2)	0.0191 (3)	0.0194 (3)	−0.0033 (2)	−0.0001 (2)	−0.0008 (2)
C30	0.0179 (2)	0.0206 (3)	0.0233 (3)	−0.0023 (2)	−0.0004 (2)	−0.0027 (2)
C1A	0.0385 (4)	0.0284 (4)	0.0274 (3)	−0.0071 (3)	0.0019 (3)	0.0013 (3)
C2A	0.0360 (4)	0.0282 (4)	0.0290 (4)	0.0004 (3)	0.0000 (3)	0.0030 (3)
C3A	0.0301 (4)	0.0350 (4)	0.0271 (3)	−0.0047 (3)	0.0005 (3)	−0.0001 (3)

### Geometric parameters (Å, º)

**Table:**

N1—C2	1.3861 (9)	C27—C28	1.3741 (11)
N1—C8	1.3892 (9)	C28—C29	1.4221 (9)
N1—C11	1.4304 (8)	C29—C30	1.4249 (10)
N3—C2	1.3180 (9)	C4—H4	0.9500
N3—C9	1.3943 (9)	C5—H5	0.9500
C2—C21	1.4798 (10)	C6—H6	0.9500
C4—C5	1.3878 (11)	C7—H7	0.9500
C4—C9	1.4022 (10)	C12—H12	0.9500
C5—C6	1.4066 (11)	C13—H13	0.9500
C6—C7	1.3903 (11)	C14—H14	0.9500
C7—C8	1.3941 (11)	C15—H15	0.9500
C8—C9	1.4055 (9)	C16—H16	0.9500
C11—C12	1.3897 (10)	C22—H22	0.9500
C11—C16	1.3939 (10)	C23—H23	0.9500
C12—C13	1.3928 (10)	C24—H24	0.9500
C13—C14	1.3892 (11)	C25—H25	0.9500
C14—C15	1.3892 (12)	C26—H26	0.9500
C15—C16	1.3912 (10)	C27—H27	0.9500
C21—C22	1.3825 (10)	C28—H28	0.9500
C21—C29	1.4286 (9)	C1A—C2A	1.3906 (15)
C22—C23	1.4131 (11)	C1A—C3A	1.3891 (14)
C23—C24	1.3708 (11)	C2A—C3A^i^	1.3876 (14)
C24—C30	1.4191 (11)	C1A—H1A	0.9500
C25—C26	1.3724 (12)	C2A—H2A	0.9500
C25—C30	1.4189 (11)	C3A—H3A	0.9500
C26—C27	1.4120 (13)		
			
C2—N1—C8	106.49 (5)	C5—C4—H4	121.00
C2—N1—C11	126.57 (6)	C9—C4—H4	121.00
C8—N1—C11	126.72 (6)	C4—C5—H5	119.00
C2—N3—C9	104.76 (6)	C6—C5—H5	119.00
N1—C2—N3	113.12 (6)	C5—C6—H6	119.00
N1—C2—C21	120.27 (6)	C7—C6—H6	119.00
N3—C2—C21	126.61 (6)	C6—C7—H7	122.00
C5—C4—C9	118.02 (7)	C8—C7—H7	122.00
C4—C5—C6	121.39 (7)	C11—C12—H12	120.00
C5—C6—C7	121.50 (7)	C13—C12—H12	120.00
C6—C7—C8	116.58 (7)	C12—C13—H13	120.00
N1—C8—C7	131.99 (6)	C14—C13—H13	120.00
N1—C8—C9	105.10 (6)	C13—C14—H14	120.00
C7—C8—C9	122.85 (6)	C15—C14—H14	120.00
N3—C9—C4	129.80 (6)	C14—C15—H15	120.00
N3—C9—C8	110.53 (6)	C16—C15—H15	120.00
C4—C9—C8	119.65 (7)	C11—C16—H16	120.00
N1—C11—C12	119.52 (6)	C15—C16—H16	120.00
N1—C11—C16	119.59 (6)	C21—C22—H22	120.00
C12—C11—C16	120.89 (6)	C23—C22—H22	120.00
C11—C12—C13	119.26 (7)	C22—C23—H23	120.00
C12—C13—C14	120.16 (7)	C24—C23—H23	120.00
C13—C14—C15	120.30 (7)	C23—C24—H24	120.00
C14—C15—C16	120.01 (7)	C30—C24—H24	120.00
C11—C16—C15	119.37 (7)	C26—C25—H25	120.00
C2—C21—C22	119.43 (6)	C30—C25—H25	120.00
C2—C21—C29	120.29 (6)	C25—C26—H26	120.00
C22—C21—C29	120.13 (6)	C27—C26—H26	120.00
C21—C22—C23	120.80 (7)	C26—C27—H27	120.00
C22—C23—C24	120.00 (7)	C28—C27—H27	120.00
C23—C24—C30	120.97 (7)	C27—C28—H28	120.00
C26—C25—C30	120.75 (7)	C29—C28—H28	120.00
C25—C26—C27	119.94 (8)	C2A—C1A—C3A	120.00 (9)
C26—C27—C28	120.78 (7)	C1A—C2A—C3A^i^	119.81 (9)
C27—C28—C29	120.63 (7)	C1A—C3A—C2A^i^	120.19 (9)
C21—C29—C28	122.80 (6)	C2A—C1A—H1A	120.00
C21—C29—C30	118.72 (6)	C3A—C1A—H1A	120.00
C28—C29—C30	118.48 (6)	C1A—C2A—H2A	120.00
C24—C30—C25	121.24 (7)	C3A^i^—C2A—H2A	120.00
C24—C30—C29	119.33 (6)	C1A—C3A—H3A	120.00
C25—C30—C29	119.42 (6)	C2A^i^—C3A—H3A	120.00
			
C8—N1—C2—N3	−0.31 (8)	C16—C11—C12—C13	−0.28 (11)
C8—N1—C2—C21	178.80 (6)	N1—C11—C16—C15	−178.15 (7)
C11—N1—C2—N3	−175.10 (6)	C12—C11—C16—C15	1.18 (11)
C11—N1—C2—C21	4.01 (10)	C11—C12—C13—C14	−0.76 (11)
C2—N1—C8—C7	−177.27 (8)	C12—C13—C14—C15	0.90 (12)
C2—N1—C8—C9	−0.10 (7)	C13—C14—C15—C16	0.00 (12)
C11—N1—C8—C7	−2.50 (12)	C14—C15—C16—C11	−1.03 (12)
C11—N1—C8—C9	174.68 (6)	C2—C21—C22—C23	−174.88 (7)
C2—N1—C11—C12	−120.91 (8)	C29—C21—C22—C23	0.72 (11)
C2—N1—C11—C16	58.42 (10)	C2—C21—C29—C28	−5.79 (10)
C8—N1—C11—C12	65.33 (9)	C2—C21—C29—C30	173.63 (6)
C8—N1—C11—C16	−115.34 (8)	C22—C21—C29—C28	178.65 (7)
C9—N3—C2—N1	0.57 (8)	C22—C21—C29—C30	−1.92 (10)
C9—N3—C2—C21	−178.47 (7)	C21—C22—C23—C24	1.44 (12)
C2—N3—C9—C4	177.86 (7)	C22—C23—C24—C30	−2.34 (12)
C2—N3—C9—C8	−0.62 (8)	C23—C24—C30—C25	−177.62 (8)
N1—C2—C21—C22	60.23 (9)	C23—C24—C30—C29	1.09 (11)
N1—C2—C21—C29	−115.36 (7)	C30—C25—C26—C27	0.42 (13)
N3—C2—C21—C22	−120.80 (8)	C26—C25—C30—C24	178.77 (8)
N3—C2—C21—C29	63.62 (10)	C26—C25—C30—C29	0.05 (13)
C9—C4—C5—C6	0.43 (11)	C25—C26—C27—C28	−0.17 (13)
C5—C4—C9—N3	−178.03 (7)	C26—C27—C28—C29	−0.58 (13)
C5—C4—C9—C8	0.34 (10)	C27—C28—C29—C21	−179.54 (7)
C4—C5—C6—C7	−0.89 (11)	C27—C28—C29—C30	1.04 (11)
C5—C6—C7—C8	0.51 (11)	C21—C29—C30—C24	1.04 (10)
C6—C7—C8—N1	177.03 (7)	C21—C29—C30—C25	179.78 (7)
C6—C7—C8—C9	0.28 (11)	C28—C29—C30—C24	−179.51 (7)
N1—C8—C9—N3	0.45 (8)	C28—C29—C30—C25	−0.77 (10)
N1—C8—C9—C4	−178.22 (6)	C3A—C1A—C2A—C3A^i^	−0.10 (13)
C7—C8—C9—N3	177.95 (7)	C2A—C1A—C3A—C2A^i^	0.10 (14)
C7—C8—C9—C4	−0.71 (11)	C1A—C2A—C3A^i^—C1A^i^	0.10 (13)
N1—C11—C12—C13	179.04 (7)		

Symmetry code: (i) −*x*+1, −*y*, −*z*+1.

### Hydrogen-bond geometry (Å, º)

Cg1, Cg2, Cg3, Cg4 and Cg8 are the centroids of the N1/C2/N3/C9/C8 imidazole ring, the C4–C9 fused benzene ring, the C11–C16 phenyl ring, the C21–C24,C30/C29 fused benzene ring and the C1A,C2A,C3A',C1A',C2A',C3A benzene ring, respectively.

**Table:**

*D*—H···*A*	*D*—H	H···*A*	*D*···*A*	*D*—H···*A*
C28—H28···N3	0.95	2.61	3.2113 (10)	121
C7—H7···*Cg*4^ii^	0.95	2.75	3.6019 (8)	150
C15—H15···*Cg*8^iii^	0.95	2.99	3.6981 (9)	132
C15—H15···*Cg*8^iv^	0.95	2.99	3.6981 (9)	132
C22—H22···*Cg*1^v^	0.95	2.91	3.6478 (8)	136
C24—H24···*Cg*3^vi^	0.95	2.76	3.4888 (9)	134
C26—H26···*Cg*2^iv^	0.95	2.87	3.5801 (9)	133
C27—H27···*Cg*1^iv^	0.95	2.97	3.7258 (8)	137

Symmetry codes: (ii) *x*+1, *y*, *z*; (iii) *x*, *y*+1, *z*; (iv) −*x*+1, −*y*+1, −*z*+1; (v) −*x*+1, −*y*+1, −*z*; (vi) *x*−1, *y*, *z*.
